# Barriers and facilitators to implementing Food is Medicine programs: Evidence from 21 food bank–healthcare partnerships

**DOI:** 10.1093/tbm/ibaf013

**Published:** 2025-05-31

**Authors:** Bailey Houghtaling, Eliza Short, Christopher R Long, Elizabeth T Anderson Steeves, Maryan Isack, Laura Flournoy, Nicole Cawrse, Elise August, Wm Thomas Summerfelt, Eric Calloway

**Affiliations:** Center for Nutrition & Health Impact, Omaha, NE, USA; Department of Human Nutrition, Foods, and Exercise, Virginia Tech, Blacksburg, VA, USA; Center for Nutrition & Health Impact, Omaha, NE, USA; Center for Nutrition & Health Impact, Omaha, NE, USA; Center for Nutrition & Health Impact, Omaha, NE, USA; Center for Nutrition & Health Impact, Omaha, NE, USA; Center for Nutrition & Health Impact, Omaha, NE, USA; Center for Nutrition & Health Impact, Omaha, NE, USA; Feeding America National Organization, Chicago, IL, USA; Rural Community Health, Western Colorado University, Gunnison, CO, USA; Center for Nutrition & Health Impact, Omaha, NE, USA

**Keywords:** food insecurity, healthy diet, access to healthy foods, health care sector

## Abstract

**Background:**

Food is Medicine (FIM) programs identify people experiencing food insecurity and diet-related chronic disease and connect them with nutritious foods. Food banks and healthcare partners are well positioned to deliver FIM programs; however, there is limited knowledge about factors that influence FIM program implementation in this context.

**Purpose:**

The goal of this study was to understand barriers and facilitators to FIM program implementation within food bank–healthcare partnerships in diverse US settings.

**Methods:**

A phenomenological study using semi-structured interviews was conducted with 21 programmatically and contextually diverse Food as Medicine 3.0 (FAM3) grantees, including food bank leads and some healthcare partners. The Consolidated Framework for Implementation Research (CFIR) 2.0 informed interview guide development, coding, and interpretation. Interviews and the analysis were completed by a team of trained researchers following best practices. Data was analyzed using Dedoose (version 9.2.12).

**Results:**

Fifty participants across 21 FAM3 grantees engaged in an interview. Most grantees shared challenges related to initiating and maintaining the healthcare partnerships needed for FIM programs. The tracking, gathering, and/or sharing of FIM program implementation and evaluation data was another primary challenge. Furthermore, limited healthcare and food bank staff capacity to carry out FIM programs was another prominent barrier. Despite these challenges, FIM programs were considered adaptable, testable, and to meet a core need among neighbors, all of which were implementation facilitators.

**Conclusions:**

Results of this study inform the need to design and test implementation strategies to overcome barriers to the implementation of a promising food bank–healthcare partnership model for FIM.

## Teaser

A qualitative study of national food bank–healthcare partnerships that help patients experiencing food insecurity and diet-related chronic disease by improving healthy food access reveals program leaders’ insights about ways Food is Medicine programs can be carried out successfully.

**Implications**

**Research:** Food bank and healthcare partnership Food is Medicine programs have shared multi-level barriers and facilitators across contexts that can be used to design and test implementation strategies.

**Practice:** Opportunities to support partnerships and staff capacity for Food is Medicine programs between food bank and healthcare settings are needed.

**Policy:** Community-based organizations like food banks need support from government and health care payers and providers to evolve a Food is Medicine system that supports their participation and recognizes their value.

## Background

Food is Medicine (FIM) programs have gained national attention as a strategy to address persisting food insecurity and diet-related chronic disease among populations with lower income in the USA [1]. FIM programs, such as medically tailored meals, medically tailored groceries, or produce prescriptions, engage the healthcare sector to promote nutrition security [2] or accessibility to enough foods and beverages that are also known to prevent, treat, or manage diet-related chronic disease among households with lower income and increased risk for diet-related conditions [3, 4]. While diverse FIM program models are being evaluated for impact throughout the USA [5–7], there has been limited attention to the factors that influence FIM program implementation across diverse contexts [8, 9]. This gap limits opportunities to inform best practices for implementing FIM programs to ensure beneficial population outcomes.

Implementation science is the science of translating evidence-based innovations, like FIM, to standard practice [10]. Implementation strategies are interventions designed to support the adoption, implementation, and sustainability of innovations for public health impact [11, 12]. As FIM programs have not been widely investigated using an implementation science framing, understanding the determinants (e.g. barriers, facilitators) to implementation across diverse US contexts is needed to inform the implementation strategies required to improve and maintain implementation [8, 9]. This is important given many FIM programs may be implemented in under-resourced community and healthcare settings that could already face challenges with organizational capacity to initiate new areas of programming [9, 13, 14]. Examples include the growing FIM program initiatives in the USA that are led by food banks, which are hunger-relief organizations that source and distribute food to households experiencing food insecurity [15], in partnership with healthcare settings [9, 13].

Initial evaluations of food bank–healthcare partner FIM programs are encouraging, given improvements to participants’ food insecurity, dietary intake, and diet-related health outcomes have been shown [7, 16]. Food banks are particularly promising as leaders of FIM programs because they have access to large quantities of healthy food, and they serve community members (i.e. neighbors) who experience high prevalences of food insecurity and chronic disease [15]. However, food banks are not equipped to address this need alone. As such, collaborative partnerships between food banks and healthcare providers to implement FIM programs have been one way to address a shared organizational goal of improving healthy food access, health, and health equity across the nation [15].

## Purpose

The goal of this study was to understand barriers and facilitators to FIM program implementation within food bank–healthcare partnerships in diverse US settings. Using the Consolidated Framework for Implementation Research (CFIR) 2.0 [17], implementation barriers and facilitators across 21 Food as Medicine 3.0 (FAM3) models funded by Feeding America and Elevance Health Foundation (described below) were explored. Results can inform future research and policy opportunities to facilitate FIM programs in the USA, as well as the implementation strategies needed to support the practice of FIM in food bank and healthcare settings.

## Methods

### Study overview

The present study was initiated in early 2023 as part of an interview process with grantees of Feeding America and Elevance Health Foundation’s FAM3 project [18]. Several co-authors (B.H., E.S., C.R.L., M.I., L.F., N.C., E.T.A.S., and E.C.) whose role on this project was external evaluation, and the goal of these interviews was to understand FAM3 grantees’ (herein: grantees) FIM program background, process, and barriers and facilitators at the start of the funding period. The methodological orientation of this study was phenomenology [19], given the goal was to explore the phenomena of implementing a FIM program by interviewing those directly involved in those activities. This study was considered exempt from human subject research oversight by the University of Nebraska Medical Center Institutional Review Board. The Consolidated Criteria for Reporting Qualitative Research [20] was used to guide reporting.

### FAM3

Feeding America, the largest hunger-relief organization in the USA, was funded by the Elevance Health Foundation, the philanthropic arm of the insurance provider Elevance, to conduct FAM3, a project designed to support and evaluate programs that identify people experiencing food insecurity and connect them with nutritious foods to facilitate better health. Food banks submitted applications for funding and were selected based on having established healthcare partnerships, readiness and capacity for evaluation, and alignment with Elevance’s service regions to develop new or expand existing FIM efforts [18]. Twenty-one grantees were selected for FAM3, with 14 being prior FAM grantees.

### Conceptual framework

The CFIR 2.0 [17] was used to understand the multi-level barriers and facilitators to FIM across diverse contexts [21]. CFIR was originally created through the combination of several “determinant” frameworks pertaining to factors recognized to influence innovation (e.g. program, practice, policy) adoption, implementation, and sustainment in the field of implementation science [21], and was updated in 2022 based on expert feedback [17]. There are five CFIR 2.0 domains, 48 associated constructs, and 19 sub-constructs oriented as barriers or facilitators to the integration of innovations (e.g. FIM) into intended settings (e.g. food banks, healthcare sites) [17]. The five domains include the *Innovation* or characteristics of the program/practice; the *Outer Setting* or the social, political, and environmental factors of the community where the innovation occurs; the *Inner Setting* or the features of organizations where the innovation is implemented; *Individuals* or the characteristics of the people responsible for the implementing; and the *Implementation Process* or the procedures used to ensure innovation adoption, implementation, and sustainment [17].

The CFIR/CFIR 2.0 is a widely used framework in implementation science [22] meant to be applicable to a variety of settings [17, 21]. CFIR 2.0 was specifically chosen for this study given its comprehensive nature, which was considered a strength for identifying contextual barriers and facilitators at multiple levels through a large-scale research evaluation that occurred across several implementation contexts, a scope that has not been previously conducted (to the best of our knowledge). Iterative adaptations were made to improve the fit and operability of CFIR 2.0 for this study. For example, terminology was modified for clarity and ease of use among the research team (e.g. “FIM Programs” versus “Innovation”); and the *Inner Setting* was defined as food bank settings, given grantees were the main focal point of interviewing efforts (i.e. healthcare partner determinants were considered “Partnerships & Connections”). Furthermore, certain CFIR 2.0 *Individuals* constructs/sub-constructs were removed based on complexity and relevancy to the study context, given it was not possible to discern who may be a *High-level Leader* versus an *Implementation Facilitator* versus an *Implementation Team Member*, for example, in a food bank setting where staff and volunteer capacity for delivering innovations is limited [14]. The CFIR 2.0 framework as applied to this study is available as a supplemental file (Supplement I).

### Role of researchers

The evaluation team members responsible for interviewing and/or analysis included B.H. (PhD-trained, female), E.S. (PhD-trained, female), C.R.L. (PhD-trained, male), E.T.A.S. (PhD-trained, female), M.I. (MS-trained, female), L.F. (MS-trained, female), N.C. (MS-trained, female), and E.C. (PhD-trained, male). Five members of the research team were also trained Registered Dietitian Nutritionists (RDN) (B.H., E.S., E.C., E.T.A.S., and N.C.). Prior to this study, all research team members had minimal contact with grantees, although they were not completely unknown given the role of the research team (external evaluation) in the Feeding America-Elevance Health Foundation FAM3 project. The authorship team is active in FIM research and practice, with the goal of understanding impact and implementation of FIM programs for improved public health and health equity.

### Sample and recruitment

The 21 grantees selected for the FAM3 project spanned 15 states and the District of Columbia and collectively included partnerships with over 50 healthcare sites. Health care partners included federally qualified health centers, academic health care systems, non-profit health care system, and others. The types of clinics within each health care system included, for example, primary care clinics, free clinics, obstetrics and gynecology clinics, cancer centers, endocrinology, in-patient hospital settings, and mental health centers. Furthermore, the 21 FAM3 program models varied across sites (Table 1). Most (*n* = 15, 71%) provided food at a healthcare partner clinic, while fewer provided food vouchers for off-site redemption (*n* = 5, 24%) or a referral to food assistance (e.g. food pantry or soup kitchens). In addition to food insecurity, some programs included other eligibility criteria to participate, such as having a diet-related condition (*n* = 6, 29%). Furthermore, most had no limit to the duration of neighbor participation (*n* = 11, 52%), while the frequency of food distribution varied among others from more (e.g. biweekly, weekly, monthly) to less structured (e.g. every clinic appointment). Program components consisted of a distribution of a wide variety of foods (e.g. fruits and vegetables, shelf-stable items), varying types of nutrition education (e.g. group class, individual visit, or education handouts, versus none/unknown), and almost all programs (*n* = 20, 95%) included federal nutrition assistance program enrollment as a wrap-around service (Table 1).

**Table 1.** Characteristics of programs implemented among Food as Medicine 3.0 (FAM3) grantees, *n* = 21^a^

FAM 3.0 programs	*n* (%)
**Program type**	
On-site clinic food distribution	15 (71%)
Food voucher for off-site redemption	5 (24%)
Clinic referral to food assistance	2 (10%)
**Population served**	
Food insecure	21 (100%)
Diet-related condition	6 (29%)
Prenatal/pediatric	2 (10%)
Other population (e.g. seniors)	2 (10%)
**Food included**	
Fruits and vegetables	15 (71%)
Other nutritious foods (e.g. low sodium)	13 (62%)
Shelf-stable items	12 (57%)
**Frequency of food distribution**	
Every clinic appointment	8 (38%)
Biweekly	5 (24%)
Weekly	4 (19%)
Monthly	2 (10%)
No standard frequency	2 (10%)
**Nutrition education components**	
Group class	7 (33%)
None or unknown	7 (33%)
1:1 visit with registered dietitian	5 (24%)
Passive nutrition education (e.g. handouts)	5 (24%)
**Wrap around services**	
Federal nutrition assistance program	20 (95%)
Other benefits support (e.g. housing)	7 (33%)
Referral to additional food bank programs	3 (14%)
Medicaid	3 (14%)
None	1 (5%)
**Program duration**	
No limit	11 (52%)
1 year	4 (19%)
Other (e.g. until end of gestation)	4 (19%)
3–6 months	3 (14%)

^a^Food bank grantees may have multiple program components; thus, total *n* for each characteristic may be >21.

All 21 grantees participated in an interview at the start of the grant funding period. An email explaining the purpose of the interview with an invitation for participation was sent to all FAM3 project leads, with a suggestion to include other food bank and/or healthcare partner staff as appropriate. All grantees agreed to participate in an interview. Fourteen interviews included multiple perspectives in addition to the FAM3 project lead. One interview session included one or more food bank staff members, and four included one or more healthcare partners. In total, 50 people participated across 21 interviews.

### Data collection

A semi-structured interview guide (supplemental file II) was created to explore FIM program implementation within the FAM3 project context. Questions were designed following CFIR 2.0 domains and to focus on understanding grantees’ implementation experiences, lessons learned, and plans for the grant period. Questions were intentionally broad, as this first interaction allowed the program and evaluation teams to become more familiar with grantee operations and plans pertaining to FIM. All interviews were carried out between February and March of 2023 by one researcher (BH, ES, CL, EAS, or EC) and a note taker. Staff from Feeding America and/or Elevance Health Foundation observed interviews to learn about grantees/FIM programs, although they did not contribute to the interview process. The Zoom web-based video conferencing platform was used to carry out and audio-record all interviews with participant consent. Interview length ranged from 16.8 to 61.5 minutes (average 45.2 minutes). No compensation was provided for participation.

### Data analysis

Prior to coding interview transcripts, coders were oriented to implementation science concepts and the CFIR 2.0 framework through lead author-recommended readings and resources. The analysis team (BH, EC, ES, MI, LF) selected an interview transcript and independently applied CFIR 2.0 codes as a training exercise, which also led the study team to modify CFIR 2.0 for use in this study, as described above. After this process, inter-rater reliability (IRR) training tests were used. The lead author, an implementation scientist in public health nutrition with prior experience using CFIR 2.0, independently coded two transcripts and two research associates (L.F. and M.I.) completed IRR training tests against these applied codes (ranging from 0.56 to 0.91 kappa statistic). Regular meetings provided opportunities for troubleshooting coding consistency; a decision tree was also created to aid coders in selecting the appropriate CFIR 2.0 domain. After acceptable IRR was established (range 0.76–0.87) [23], all 21 transcripts were divided and coded by MI and LF. Then, BH reviewed all code applications and made modifications when necessary. All coding and analysis procedures occurred in Dedoose (version 9.2.12, Los Angeles, CA: SocioCultural Research Consultants, LLC).

For thematic analysis, results by CFIR 2.0 domain/construct were first reviewed by BH to understand if barriers/facilitators to FAM3 programs varied among grantees with higher versus lower intensity programs (e.g. those with more frequent neighbor touchpoints, comprehensive nutrition education, and added services versus those with one-time food box service). No prominent differences in CFIR 2.0 results were observed; therefore, results were analyzed and narrated in aggregate. Concepts that were only described once regarding CFIR 2.0 code application were not reported. Furthermore, qualitative data saturation was not assessed, given project resources allowed for one interview per FAM3 project (versus multiple follow-up interviews). Finally, a member checking process was used to ensure the results aligned with grantee experiences as program implementors, which included a presentation of results with opportunities for discussion. No changes to the results were indicated from this process.

## Results

Following, the barriers and facilitators to FIM programs (*n* = 50 participants across 21 grantees) are narrated. Note, the term FIM is used broadly in the Results section, given many grantees had been implementing different iterations of FIM programs over time and their perspectives reflected general learnings both from FIM initiatives broadly and from FAM3 projects specifically. Example quotations as well as the number of code applications for each CFIR 2.0 domain/construct are shown in Table 2. Results are ordered by CFIR 2.0 domain/construct based on the prominence of information shared by participants. Lower prominence results shared by one-third or fewer (i.e. 7 or less) grantees are described only in Table 2. This decision was intended to draw readers’ attention to the more salient concepts in the results narrative, while still reporting all findings of interest to the research objective.

**Table 2.** Barriers and facilitators to the implementation of Food is Medicine (FIM) programs among 21 Food as Medicine 3.0 (FAM3) grantees^a^ following the Consolidated Framework for Implementation Research (CFIR) 2.0

CFIR 2.0 domains and constructs Codes/contributors	Description	Example quotation
**Outer setting**		
-Partnerships & Connections 67 codes among 21 grantees	A shared mission of addressing social determinants of health and diet-related chronic disease, while underscoring the patient benefit and business case for FIM programs, was described to drive partnerships. However, partnership development and maintenance were described to need a lot of time, and healthcare partners may need resources to distribute food to neighbors on-site. Open and honest communication was an important ingredient noted for successful partnerships.	How do we make this as patient focused as possible? But knowing things don’t stick and stay unless they make financial sense. And I really want this not to be a fleeting area of interest. I’m so interested in how do we really help to make sure there is sustainability longer term for all parties so that it’s something that people can count on. [Grantee 02]
-Financing 17 codes among 10 grantees	External sources of funding (e.g. grants, city funds, fundraising dollars) were key for supporting the longevity of FIM programs, program infrastructure, higher quality food items, and program expansion. The ability to reimburse for Registered Dietitian Nutritionist (RDN) services was described as a program facilitator, but not being able to reimburse for cooking classes or food purchases was a noted program barrier.	So, for a while, we had it as a pilot project because we’ve been doing it now for upwards of five years. We’ve switched from it being a project to a program. So, it is one of our designated programs, and I feel like this funding supports the whole program. [Grantee 09]
-Critical incidents 11 codes among 7 grantees	The COVID-19 pandemic burdened FIM program operations, healthcare partner capacity, and opportunities for evaluation, with long-lasting impacts. However, the COVID-19 pandemic was also described to generate awareness of diet-related chronic disease disparities and to accelerate food distribution models, both program facilitator.	During COVID, every partner had to make shifts in the way they were operating. [Grantee 09]
**Implementation process**		
-Reflecting and evaluating 116 codes among 21 grantees	Engaging healthcare partners in FIM program data tracking and sharing capabilities was described as difficult, and changes to healthcare site procedures were noted to be burdensome and costly. Ensuring electronic medical record (EMR) alignment to track and evaluate FIM programs can help, although it requires support. Closed-loop systems are also needed to minimize the burden of manual tracking and to allow for an understanding of program delivery. Furthermore, clinical data may not always be the most reliable, and it is difficult to get a good response rate for neighbor surveys.	With this program, we haven’t really had any data sharing challenges because we have access to the EMR. I think that that has to be established in the beginning as well. If you don’t have that, it’s going to be running around trying to connect with other people to be able to get data. When really, it’s all available in one spot. [Grantee 16]
-Implementation strategies 23 codes among 14 grantees	Guides, toolkits, templates, and peer learning were described as opportunities to help food bank personnel engage with healthcare partners. Provision of trainings (e.g. food insecurity, nutrition education), technical assistance, and neighbor-facing materials on food resources to healthcare partners was also considered useful to support FIM program delivery in healthcare sites.	…but we do come in to support and there are times when myself or my team may come in and … override some of the things that’s happening because again, we want it to move smoothly. We want it to be dignified and so we may step in just a little to sort of steer it … but it’s a combined effort. I always say that we cannot do it without the health sites and their team and the health sites will say the same thing. [Grantee 06]
**Individuals**		
-FIM deliverers 52 codes among 17 grantees	Staffing and staff capacity to support FIM programs were described as limited at healthcare and food bank sites. Although having a designated support person to assist with connecting neighbors to program and community services was noted as key for engagement. Program “champions” were also described as necessary for successful FIM programs at each site.	That, but they’re nurses and doctors and they’re busy. I mean, we see the same thing with schools. For instance, with our backpack program, it’s hard to get those teachers to do extra stuff. I mean, they’re busy. So I think it’s that same premise of that it’s just something else that they have to do. So we just have to find the right person in their clinic to do it. [Grantee 11]
-FIM recipients 39 codes among 19 grantees	FIM programs were considered to meet a core need among neighbors who struggle with food and nutrition security and meeting basic needs. Transportation, access, and program eligibility criteria barriers were noted to limit neighbor engagement with FIM programs and to require unique strategies to overcome.	…it all starts with food. That’s the number one thing that they needed was that. Once they got that out of the way, they were able to focus on other things. But I have seen it, I’ve seen many people’s lives change after they come talk to us they get set up with food… [Grantee 20]
-Leadership 14 codes among 9 grantees	Supportive higher-level (e.g. CEO) and physician leadership for implementing FIM programs was linked with program adoption and staff motivations for program delivery.	The CEOs met with our CEO and it sort of trickled down. It was the CEO was super excited about it and then the staff became super excited about it. And so it became a model that everyone wanted to be a part of… [Grantee 06]
**FIM programs**		
-Adaptability 37 codes among 17 grantees	The ability to modify aspects of FIM programs was a facilitator to improve neighbor reach and engagement; program services; and to respond to changes in the environment, such as from natural disasters. Changes to FIM programs included changing food delivery modes, eligibility criteria, service sites, and the types of foods provided, for example.	I’m excited to get access to people who would otherwise go without the services, for whatever reason. I think that there are pockets of people we can’t reach unless we go to where they are. [Grantee 18]
-Trialability 22 codes among 13 grantees	Testing FIM programs at a smaller scale, both to understand effectiveness and to learn from implementation was considered beneficial for fine-tuning program design and neighbor engagement strategies, improving staffing capacity and confidence, and developing strong partnerships before expansion.	So that makes it a whole lot easier to go forward with a little bit of confidence and say like, “Hey, this program works, and we will try to scale it.” [Grantee 18]
-Design 11 codes among 8 grantees	FIM programs were described as designed to support neighbors’ needs and improve the likelihood neighbors will engage in program services (e.g. cultural appropriateness). Programs also often included referrals to other federal assistance programs, in addition to the provision of food, to help with meeting basic needs that are not covered by any one program.	We encourage people to apply for SNAP [Supplemental Nutrition Assistance Program], because we know that our food programs are not enough, and SNAP is not enough on its own. [Grantee 20]
-Source 4 codes among 4 grantees	Co-creating FIM approaches between food banks, healthcare, and other partners was described as a valuable approach. Having models to draw from, such as Feeding America’s FIM structure, was also noted as helpful.	So, while we were going on with those conversations [securing healthcare partnerships], we were researching what other people were doing in the community, and [FAM programs] were fairly new, there weren’t a lot to go off of, so we kind of gleaned from that information, as well as just the pantries that we already operate. We brought in our dietitian to come and advise us on the types of food, as well as our food procurement team to the types of food that we could secure. [Grantee 03]
-Cost 3 codes among 3 grantees	Food purchasing for FIM programs was described as costly and was sometimes limited by vendor policies for bulk purchasing. Medically tailored meal programs were also discussed as more costly than FIM programs that use donated food models.	Because we rely on all purchased food, it is not our typical, we have had to bring in new vendors. We have to change the way that we run our systems, and it’s a lot more costly and a lot smaller scale in some instances…. Because it’s all purchased food, and we don’t use any other types of food, it’s very expensive. [Grantee 02]
**Inner setting**		
-Relational connections 4 codes among 3 grantees	Proximity to internal and external human resources aligned with the goals of FIM programs was a facilitator. This included office locations in close proximity to other working public health professionals (e.g. WIC, SNAP screeners) and working with internal operations departments to handle food procurement.	I’m sure that the other food banks have those connections, but a connection of, “Hey. I know how to reach out to this person,” versus, “I can literally stumble into this person,” is a little bit different. [Grantee 14]

^a^Feeding America and Elevance Health Foundation’s FAM3 mechanism funded 21 food banks with strong healthcare partnerships, readiness and capacity for evaluation, and alignment with Elevance’s markets to develop or expand FIM efforts.

### Outer setting

#### Partnerships and connections

A shared goal of addressing social determinants of health and prevalent diet-related chronic diseases between food bank and healthcare sectors was described by many. This mission alignment was considered key for developing and maintaining strong food bank–healthcare partnerships for FIM programs. Leveraging the patient-focus and “business case” of FIM programs to healthcare partners was a recommendation for framing the importance of FIM partnerships to minimize potential push-back. Skill development regarding communicating with healthcare partners was another need, given healthcare terminology was referred to as “a different language.” Other challenges of establishing and maintaining healthcare partnerships were the need for varied approaches among different clinical sites/partners and time constraints, given significant time was required for in-person communications (especially new partnerships) and for navigating healthcare legal agreements.

Food bank personnel often described providing infrastructure like refrigeration to support on-site fresh food distribution for FIM programs at healthcare sites, although it was noted that limited clinic space at some sites may prevent this approach. In these instances, FIM programs were described to focus on pre-packaged and shelf-stable foods, although these items still required (less permanent) space for storage. Grantees mentioned that healthcare partners were also sometimes hesitant to offer on-site food distribution models due to not wanting to be seen as a food access organization. However, on-site clinic food distribution models were acknowledged to improve the redemption of services. Ultimately, grantees acknowledged the need to align food distribution options with clinic capabilities and interest.

Grantees also described healthcare partners as having heard about successful FIM programs by word-of-mouth or through mutual partners. As such, potential healthcare partners sometimes approached food banks to participate in FIM programs and, while positive, new partnerships were considered a challenge if FIM programs were still developing infrastructure for quality program delivery and were not yet ready to expand. Regarding food bank–healthcare partnership interactions, transparent and bidirectional communication were considered key ingredients for strong partnerships, including space to communicate implementation pain points, opportunities for improvement, and/or needs and expectations.

Furthermore, the broader partnerships that grantees engaged in FIM programs were described to be informed by neighbor needs (e.g. housing) and were ideally complementary (e.g. an established food delivery services), rather than attempting all facets of an FIM program as a single organization. Being proactive about attending community events to advocate for food and nutrition was also described as an opportunity to develop new partnerships. Last, a fear of violating Health Insurance Portability and Accountability Act (HIPAA) policies, as well as the newness of FIM programs relative to the complex asks, like sensitive data sharing, was a deterrent for healthcare partnerships shared among some grantees.

#### Financing

While all grantees were supported by external funding at the time of this study, some specifically called out financing as a facilitator to FIM programs. The Feeding America FAM grants, as well as other sources of grant dollars, allowed grantees’ projects to evolve into long-term FIM programs offered by food bank organizations. Grant funding supported FIM program partnership development and maintenance; innovation and specialization (e.g. high-risk pregnant moms); expansion; food services improvements; capacity building; and incentive money for participant surveying. Local sources of funds (e.g. city) and fundraising were also described by grantees to support FIM program infrastructure; connections with local community partners; and food purchasing. Also, the ability to reimburse for RDN services was a facilitator to FIM programs, but the inability to reimburse for other services like cooking classes or food purchasing were barriers. Last, the need to generate financial support from managed care organizations or insurers was important for FIM program sustainability from grantees’ perspective, although ensuring local community-based organizations still “fit” into the broader FIM landscape was a noted concern.

### Implementation process

#### Reflecting and evaluating

Conversations between food bank personnel and healthcare partners on data tracking and sharing regarding FIM program referrals, utilization, and health impact were described as difficult (and partially attributed to HIPAA compliance concerns). At some healthcare sites, data tracking and sharing procedures were considered less of a priority due to the perceived difficulty of engaging healthcare partners in the consistent screening and referral of neighbors to FIM program services. However, grantees noted that remaining flexible and solution-oriented were facilitators for FIM program data tracking and sharing in the healthcare context. Reaching agreement regarding the patient-level data that should be tracked to indicate program success was another need/facilitator, including focusing these conversations on the data healthcare partners already tracked.

Using electronic medical record (EMR) technology was described as a streamlined approach to tracking healthcare data in relation to FIM program outcomes. Grantees often described EMR technology integration as the goal for FIM programs, although they acknowledged the associated cost and burden and, thus, the value of incremental changes to existing EMR systems. Changes to EMR capabilities for data sharing were considered a challenging new “ask” for healthcare partners, who were described to be still learning about FIM programs during implementation. Grantees also mentioned that healthcare sites within a similar region may use different EMR systems, which was another challenge to FIM program data tracking and sharing across partner sites. Working with a data analytics team and having access to information technology (IT) support were both described as facilitators, as well as engaging external academic teams to support EMR data tracking/sharing/analysis. Short-term volunteers (e.g. volunteers, students) for neighbor surveys were also considered a facilitator among some grantees.

Ultimately, grantees described a closed-loop data tracking and sharing process as both a facilitator and a need to limit implementation burden and to improve process (e.g. screening and referral, utilization) and outcome knowledge for FIM. Available case management software platforms were described by grantees to help bridge data sharing gaps, demonstrate program value, and improve neighbor outcomes through informed program tailoring opportunities. Although, limitations to these platforms were also described by grantees, including limits to the number of outcomes that could be tracked and capacity limitations among clinic partners, especially if requiring added procedures. While clinical data (e.g. hemoglobin A1c) were described as an attractive evaluation opportunity, it was also acknowledged that these data were not always high quality, reliable, or useful for FIM program timelines. Specific evaluation protocols for gathering clinical outcomes outside of standard care procedures were an opportunity for more accurate clinical data from the perspective of some grantees.

In addition to FIM program outcomes, grantees also wanted opportunities to evaluate healthcare partners’ and neighbors’ experiences delivering and participating in FIM programs, respectively. Neighbor evaluation surveys, a core component of FAM3, were considered challenging to collect, even with incentives, and especially if longer in length (e.g. 20 minutes). Grantees also described paper survey options as facilitators for evaluation, given technologies like QR codes and online surveys were unreliable at times and across sites (e.g. incompatible technology, internet connectivity issues). Furthermore, because of the holistic focus of many FIM programs, some grantees described difficulty in choosing the right measures for evaluating program success, with quality-of-life metrics described as a potential favorable option. Some grantees also described that return-on-investment data, while attractive to organizational leadership and other invested parties, was challenging and often not feasible to collect and report.

#### Implementation strategies

Grantees described added supports that could help with FIM program implementation, including guides, templates, or checklists for healthcare partner engagement (e.g. conversation tips, FIM program asks); toolkits with implementation materials, resources, or lessons learned; and opportunities for peer learning. Grantees also described providing some implementation support to FIM program partners, including training for healthcare partners on topics such as food safety, food insecurity, nutrition education, and screening/referral procedures. Furthermore, efforts to build FIM program partners’ capacity and confidence to address neighbor needs were described as an important need. Regular meetings, email communication, and on-site technical assistance were additional FIM program implementation supports that some grantees mentioned providing to healthcare partners. A smaller number of grantees also described using formal partnership blueprints or toolkits to detail partnership structures, program roles and expectations, and to provide tailored implementation resources. Flyers that detailed local food distribution sites were also sometimes provided to healthcare partners. Furthermore, program branding (e.g. on bags, vehicles) and signage strategies were also used at times to raise FIM program awareness and to serve as program delivery reminders for healthcare partners. Last, some grantees described using choice architecture strategies within healthy food pantry models to nudge neighbors toward healthier food items.

### Individuals

#### FIM deliverers

Limited staffing and staff capacity for implementing FIM programs were considered barriers. Most often, challenges were described regarding the healthcare context, with clinics described as short-staffed, overburdened, and struggling with high staff turnover and difficulties in finding replacements. As FIM programs were an added ask to healthcare partners’ full-time jobs, staffing issues were considered an additional barrier to consistent referral or any added services (e.g. surveying patients), including data sharing. Engaging non-physician healthcare staff was a possible solution that grantees posed for generating more FIM program referrals. Food bank sites were also described by grantees to experience similar challenges with staffing and capacity, and, across program sites, this reality was described to limit opportunities for FIM program growth and expansion.

Although mentioned less frequently, grantees shared that having a specific person (e.g. case manager, coordinator, navigator, or community health worker) or team to provide individualized help and to connect neighbors to FIM program resources, either in person or virtually, and during times that work for neighbors (e.g. Saturdays), was a facilitator for FIM program engagement. “Champions” of FIM programs at healthcare, food pantry, and partner sites, or in each community where FIM programs were being implemented, were also described by grantees to facilitate FIM program delivery, neighbor engagement, and program evaluation. Interns and volunteers located at both food bank and healthcare sites were also described to facilitate FIM program implementation, although some grantees acknowledged that organizing volunteers required additional resources from an already limited internal staff.

#### FIM recipients

Grantees described FIM programs as meeting an enormous need for quality food and auxiliary services (e.g. diapers, COVID-19 tests, vitamins, basic-need referrals) among neighbors with heightened barriers to food and nutrition security and risk for diet-related chronic disease. However, transportation, limited pantry hours, and distance to pantries were considered barriers to neighbor engagement from grantees’ perspective. FIM programs that were designed based on knowledge of neighbors’ individual-level barriers and that used tailored referral options to accommodate needs were considered beneficial. On-site provision of food services at clinics or via locations that were highly accessible (e.g. lockers, neighborhood pantries with grocery store aesthetic) were also facilitators to neighbor engagement, as were efforts to address transportation barriers, such as by providing rideshare fares. Last, grantees described that the COVID-19 pandemic exacerbated the need for healthy food access among neighbors and that the negative effects of the pandemic have lingered. However, the COVID-19 pandemic was also described as a facilitator to some degree. For example, the public health crisis helped to raise public/partner awareness of the importance of healthy food access for health and well-being and helped to demonstrate the value of investing in home food delivery services.

Grantees also named other barriers to neighbor engagement with FIM program services, including a hesitancy to disclose personal health information about diet-related chronic diseases, which were used for program eligibility criteria. Thus, expanding eligibility criteria to reach a greater number of neighbors was considered a potential facilitating strategy. Furthermore, stigma surrounding food assistance in more rural areas was another barrier to neighbor engagement. Some grantees also shared that outreach efforts could help to improve neighbor engagement, but that these efforts were challenging. Nutrition education was also considered beneficial and necessary to improve dietary practices alongside improved healthy food accessibility, and was also considered by some grantees as the key component that differentiated FIM programs from other food access/assistance strategies. Last, some grantees considered the link between mental health and diet-related chronic disease as an important area for potential healthcare expansion (e.g. mental health providers) to meet neighbor needs.

#### FIM leadership

Leadership support and buy-in were described by grantees as a FIM program facilitator, both within food bank and healthcare settings. Higher-level leadership (e.g. Chief Executive Officer, board members) or physician buy-in were attributed to the reasons FIM programs were initiated and staff were motivated to deliver them.

### FIM programs

#### Adaptability

The ability to modify FIM programs was a facilitator. Most commonly, grantees described modifying how food benefits could be accessed to better reach neighbors, including through deliveries, pop-ups, or fixed locations with extended hours. Grantees also described changes to FIM program eligibility criteria, healthcare delivery sites, and program duration to improve reach and service, program engagement, and to maximize program health impacts. More generally, the need to adapt FIM programs was based on new learnings, external events (e.g. COVID-19), and site-level needs. Last, some grantees mentioned tailoring food resources to diet-related chronic disease requirements or allergies versus standard food provisions.

#### Trialability

The trialability of FIM programs was also described as a facilitator among some grantees. For example, testing out FIM programs prior to expansion, both to understand effectiveness and to learn from implementing programs at a smaller scale, was considered beneficial for fine-tuning design and participant engagement strategies, improving staffing capacity and confidence, and developing strong partnerships. The success of larger, successful programs was attributed to this type of approach to FIM program design and implementation.

#### Design

FIM program design was another facilitator. Primarily, FIM programs were described by grantees as designed to meet neighbors’ needs, such as through providing specific delivery or pickup options, including helping neighbors to navigate available transportation options. Ensuring food items were aligned with neighbor preferences and culture were also common design approaches to ensure program relevance and likelihood for engagement. FIM programming was also described to include referrals to other federal or local assistance programs (e.g. nutrition assistance, housing support, preventative services), given grantees acknowledged that core FIM program components could not meet all of neighbors’ basic needs. The co-design of FIM programs with healthcare or other partners was another noted facilitator for success. Last, some grantees considered the provision of nutrition education from RDN partners as a key aspect of programming that further specialized FIM programs within broader food access interventions.

## Discussion

A comprehensive implementation science framework (CFIR 2.0) [17] was used to analyze barriers and facilitators to FIM programs from the perspective of food bank grantees funded to implement FAM3 projects in partnership with healthcare sites. This work (to the best of our knowledge) is the first assessment of FIM program implementation determinants for food bank–healthcare partnerships occurring across contextually and programmatically diverse sites. The main findings from this study highlighted the challenges of initiating and maintaining the healthcare partnerships needed for FIM programs; tracking, gathering, and/or sharing FIM program implementation and evaluation data; and limited healthcare and food bank and staff capacity to carry out FIM programs. Despite these challenges, FIM programs were considered adaptable, testable, and to meet a core need among neighbors.

The primary challenges to FIM program implementation within food bank–healthcare partnerships as identified in this study mirror the challenges identified in a narrative review specific to FIM programs within healthcare settings [9]. Healthcare–community linkages beyond those for FIM program goals have been reported as challenging in general due to power imbalances, coordination challenges, and data sharing limitations [24–27]. While implementation science framing is lacking in FIM research and practice efforts in general [8, 9], the overlap in barriers and facilitators identified to date indicates there is enough information to move toward designing and testing implementation strategies [11] to address key challenges in FIM. While some grantees reported using implementation strategies to support FIM programs in this study, mainly trainings, little is known about which combination of implementation strategies are used across settings, how they are carried out, and what impacts they have.

Therefore, future directions for FIM implementation research and practice should center on naming, defining, and specifying (i.e. actor, action, action target, temporality, dose, target implementation outcome, and justification [11]) the implementation strategies used to overcome FIM healthcare partnership and program implementation barriers [8, 9, 12, 28]. Available implementation strategy compilations, such as the Expert Recommendations for Implementation Change (ERIC) [12] for clinical settings and the Implementation Strategies Applied in Communities (ISAC) for food bank settings [28], offer a starting point for selection. More attention to the creation and evaluation of implementation strategies used to strengthen FIM programs implemented within food bank–healthcare partnerships could help to maximize program efficiency and funding investments. At the same time, the field may also benefit from additional efforts to identify contextual barriers and facilitators to FIM in food bank and/or healthcare settings that are substantially different than the settings covered within this study, such as Tribal Health systems, or in investigations that emphasize food bank inner setting determinants more [29, 30].

Results of this study also have implications for FIM policy. Some grantees shared challenges or concerns related to FIM program funding and sustainability. The current policy moment in which US states, through Medicaid 1115 demonstration waivers and healthcare payers, are seeking to expand reimbursement opportunities for provision of FIM services [31] could be a viable support for FIM programming within food bank–healthcare contexts. However, reimbursement for FAM3 grantees through such mechanisms may be complicated by the reported barriers, which included initiating healthcare partnerships (e.g. contracting), accessing sustainable funding for FIM efforts (e.g. billing, reimbursement), and working with sensitive healthcare data (e.g. data infrastructure) [4, 9, 32]. To mitigate these difficulties, FIM-engaged community-based organizations like food banks will likely need additional implementation support from government, health care payers and providers, and others to evolve an FIM system that supports participation of community-based organizations and recognizes their value.

### Strengths and limitations

A notable strength of this study was the diverse sample of grantees who engaged in interviews regarding FIM program approaches, neighbor audiences, prior FIM program experience, and geography. As such, results of this study provided insights into barriers and facilitators to FIM programs implemented within food bank–healthcare partnerships that can be used to design and test solutions that are likely to be of value across diverse contexts. Use of the comprehensive implementation science framework CFIR 2.0 was also a strength that could allow for study results to be used to inform a tailored version of Robinson and Damschroder’s pragmatic context assessment tool (pCAT) [30]. For example, a FIM pCAT could aid in a rapid understanding of site-level barriers or facilitators to FIM programs for food bank and healthcare partnerships that are either external or new to Feeding America’s FAM network.

However, there are several study limitations that should be considered. Healthcare partner representatives inconsistently participated in interviews. As healthcare partners’ participation was decided by food bank grantee leads and/or was challenged by busy schedules, information pertaining to FIM program barriers from the healthcare perspective could be underrepresented. Furthermore, the attendance of FAM3 funder representatives during the interviews could have influenced grantees’ interview responses. Nonetheless, rich information about barriers and facilitators to food bank–healthcare partner FIM programs were discussed, which may have been due to many grantees’ having strong prior working relationships with the funder (Feeding America). This study also occurred at the start of the FAM3 funding cycle. While many grantees had prior experience implementing food bank–healthcare FIM programs, more research is needed to understand if and how barriers and facilitators change at various phases of FIM program implementation, especially as the sustainment of innovations is understood to be dynamic [33]. Last, interviews were limited by time, and the CFIR 2.0 is a large framework with many concepts. Grantees in this research shared fewer insights on FIM program barriers and facilitators in relation to food bank organizational factors (Inner Setting), which may warrant future research.

## Conclusion

As nationwide interest in FIM programs grows, understanding the factors that influence the success of FIM programs implemented within food bank–healthcare partnerships is critical. These insights can be used to inform the implementation strategies needed to ensure favorable FIM program impacts among populations experiencing disproportionate rates of food insecurity and diet-related chronic disease. Results of this study indicated future directions to design and test implementation strategies are required to overcome challenges with initiating and maintaining healthcare partnerships, tracking, gathering, and/or sharing FIM program implementation and evaluation data, and building healthcare and food bank staffing capacity to carry out FIM programs. Similarly, elevating the positive aspects of FIM programs identified within these contexts, such as FIM program adaptability, trialability, and fit with neighbor populations, are key facilitating strategies that could also be operationalized. Research, practice, and policy-based evidence are all needed given the dearth of information available in the literature pertaining to implementation strategies used to support FIM program adoption, implementation, and sustainability within food bank–healthcare partnerships.

## Supplementary Material

ibaf013_suppl_Supplementary_File_1

ibaf013_suppl_Supplementary_File_2

## Welfare of Animals

This article does not contain any studies with animals performed by any of the authors.
