# Supplementary material for: Barriers and facilitators to implementing Food is Medicine programs: Evidence from 21 food bank–healthcare partnerships
Source: Transl Behav Med. 2025 May 31;15(1):ibaf013. doi: 10.1093/tbm/ibaf013 (PMC12169342; doi:10.1093/tbm/ibaf013)
Supplement: ibaf013_suppl_Supplementary_File_1 [file ibaf013_suppl_supplementary_file_1.docx]

**Supplemental File I**

| **Supplemental Table.** Consolidated Framework for Implementation Research (CFIR) 2.0 for Food Bank-Healthcare Partnership Food is Medicine (FIM) Programs. | |
| --- | --- |
| **CFIR 2.0 Domain/Subdomain** | **Definition/Example** |
| **FIM (i.e., Innovation) Domain**    Definition: Use codes in the FIM domain if interview participants are sharing attitudes, beliefs, or perceptions about FIM interventions in general, or their own FIM intervention or components. This could include favorable or unfavorable perceptions about the ease of use, how it can be used, why it was chosen, etc. | |
| FIM Source | Definition: The group that developed and/or visibly sponsored FAM interventions is reputable, credible, and/or trustable. Include guidance from other groups or existing models.    Example: FIM experts are trusted and/or assisted with developing the model used by the interviewee. |
| FIM Evidence-Base | Definition: FIM interventions have robust evidence supporting its effectiveness.    Example: There was not a lot of evidence supporting efficacy of FIM models, so that posed a challenge in making a case to pursue them. |
| FIM Relative Advantage | Definition: FIM interventions are better than other available interventions or current practice.    Example: To do what they wanted to do (e.g. promote healthy diets and reduce disease risk), FIM was the best option available. |
| FIM Adaptability | Definition: FIM interventions can be modified, tailored, or refined to fit local context or needs.    Example: Changed program to better fit a specific clinic context (physical capacity)/patient population. |
| FIM Trialability | Definition: FIM interventions can be tested or piloted on a small scale and undone.    Example: They didn’t need to go all in to try FIM, they could test drive it first. So, they decided to pursue FIM. |
| FIM Complexity | Definition: FIM interventions are complicated, which may be reflected by its scope and/or the nature and number of connections and steps.    Example: FIM models were not very complicated, so they decided to pursue it. |
| FIM Design | Definition: FIM interventions are well designed and packaged, including how it is assembled, bundled, and presented.    Example: There lacked ready-made packaged models for FIM, which posed a challenge to pursuing FIM. |
| FIM Cost | Definition: FIM intervention purchase and operating costs are expensive.    Example: The operating costs of FIM were acceptable, which made it easier to make the case for FIM. |
| **Outer Setting Domain**    Definition: Use codes in the Outer Setting domain if interview participants are talking about factors or circumstances that influence FIM processes that are external to the food bank. This could include external community, region, state, national or social / political factors. | |
| Critical Incidents | Definition: Large-scale and/or unanticipated events disrupt the Outer Setting during implementation and/or delivery of the FIM program.    Example: COVID-19 disrupted our ability to screen patients for food security. |
| Local Attitudes | Definition: Sociocultural values (e.g. shared responsibility in helping recipients) and beliefs (e.g. convictions about the worthiness of recipients) encourage the Outer Setting to support implementation and/or delivery of FIM program.    Example: Attitudes, perceptions, and beliefs of the population within the outer setting – example: community members having a negative perspective of the intervention you are offering. |
| Local Conditions | Definition: Economic, environmental, political, and/or technological conditions enables the Outer Setting to support implementation and/or delivery of FIM programs.    Example: About social/environmental context of community (everything outside the food bank or health care setting) – more tangible things (e.g. to do a program, it needs to make sense economically, politically, need technology available). |
| Partnerships & Connections | Definition: The Inner Setting is networked with external entities, including referral networks, academic affiliations, and professional organization networks.    Example: We, at the food bank, had trouble getting our healthcare partners to buy-in and commit staff to the FIM project in the beginning due to their budgetary cuts, but that is no longer an issue. |
| Policies & Laws | Definition: Legislation, regulations, professional group guidelines and recommendations, or accreditation standards support implementation and/or delivery of FIM programs.    Example: Referring to local and/or community laws in place. |
| Financing | Definition: Funding from external entities (e.g. grants, reimbursement) is available to implement and/or deliver FIM programs.    Example: The ample available funding for FIM allowed us to operate the program and fund partners’ efforts. |
| External Pressure | Definition: External pressures drive implementation and/or delivery of FIM programs.  *Note: Use this construct to capture themes related to External Pressure that are not included in subconstructs.* |
| Societal Pressure | Definition: Mass media campaigns, advocacy groups, or social movements or protests drive a need to implement and/or deliver FAM programs.    Example: Increased awareness of FIM programs makes it easier for us to open conversations about it with potential partners. |
| Market Pressure | Definition: A need to compete with and/or imitate peer entities drive implementation and/or delivery of FIM programs.    Example: All the food banks in surrounding counties have implemented FIM programs, so we felt we needed to as well. |
| Performance-Measurement Pressure | Definition: Quality or benchmarking metrics or established service goals drives implementation and/or delivery of FIM programs.    Example: We get funded based on the amount of food we disseminate, so we saw FIM as another avenue for distributing food to a new group. |
| **Inner Setting Domain**    Definition: Use an Inner Setting code when interview participants describe factors within the food bank that influence FIM implementation. This includes things like the work processes and structures/roles, physical or virtual spaces, and organizational culture and connectedness. | |
| Structural Characteristics | *Note: Use this construct to capture themes related to Structural Characteristics that are not included in subconstructs.* |
| Physical Infrastructure | Definition: Layout and configuration of space and other tangible material features supports implementation and/or delivery of FIM programs.    Example: We had room and refrigeration to store FIM food prior to delivery. |
| Information Technology Infrastructure | Definition: Technological systems for tele-communication, electronic documentation, and data storage, management, reporting, and analysis supports implementation and/or delivery of FIM programs. Use this for data sharing issues related to software/hardware.    Example: We integrated our referral tracking software with our healthcare partner, which allowed us to easily enroll and prepare programming for incoming participants. |
| Work Infrastructure | Definition: Organization of tasks and responsibilities, within and between individuals and teams, supports implementation and/or delivery of FIM programs. Use this code for coding intra-team issues related to coordinating roles and responsibilities but use B. Relational Connections if the issue is across two or more teams within the same organization.    Example: Within the team that handled FIM food deliveries, we were understaffed, and sometimes tasks were missed. |
| Relational Connections | Definition: Formal and informal relationships, networks, and teams within and across internal work teams (e.g. structural, professional). Use this code for coding across team issues such as coordinating roles and responsibilities (and related issues) but use 3. Work Infrastructure for intra-team issues.    Example: Our FIM team was ready to deliver food to referred participants, but the delivery team was not notified of this a head of time and didn’t have a process for integrating our orders into their existing processes and workflow. |
| Communications | Definition: Formal and informal information sharing practices support implementation and/or delivery of FIM programs. Use this code for the processes and/or approaches related to communicating (can double code with Information Technology Infrastructure if referring to a piece of software/hardware).    Example: Our FIM team did not have the ability to schedule deliveries with the delivery team for the first several months until we were added to their scheduling system so that we could formally communicate our delivery needs. |
| Culture | *Note: Use this construct to capture themes related to Culture that are not included in subconstructs. This can be formal values (not goals/objectives) enforced by the organization or can be informal aspects of the organic work culture fostered there.* |
| Human-Equality Centeredness | Definition: There are shared values, beliefs, and norms about the inherent equal worth and value of all human beings. |
| Recipient-Centeredness | Definition: There are shared values, beliefs, and norms around caring, supporting, and addressing the needs and welfare of recipients. |
| Deliverer-Centeredness | Definition: There are shared values, beliefs, and norms around caring, supporting, and addressing the needs and welfare of deliverers. |
| Learning-Centeredness | Definition: There are shared values, beliefs, and norms around psychological safety, continual improvement, and using data to inform practice. |
| Tension for Change | Definition: The current situation is intolerable and needs to change. |
| Compatibility | Definition: FIM programs fit with workflows, systems, and processes.    Example: It was easy to add the FIM referrals to our workflow since we already had a system in place to manage this. |
| Relative Priority | Definition: Implementation and delivering FIM programs are important compared to other initiatives.    Example: Our leadership prioritized our FIM program when budget cuts happened. The FIM programming was spared, and another program was discontinued. |
| Incentive Systems | Definition: Tangible and/or intangible incentives and rewards and/or disincentives and punishments support implementation and delivery of FIM programs. Among other things, use this code for administrative/organizational policies and procedures that impact FIM activities.    Example: All the front-line workers that completed their FIM training within the first two weeks were given a small bonus. |
| Mission Alignment | Definition: Implementing and delivering FIM programs are in line with the overarching commitment, purpose, or goals of Feeding America/Health Care Partner.  *Note: This would differ from Culture in that these are about measurable goals/objectives of the food bank or commitments made by the food bank.* |
| Available Resources | *Note: Use this construct to capture themes related to Available Resources that are not included in subconstructs.* |
| Funding | Definition: Funding is available to implement and deliver FIM programs.    Example: Internally sources/internal issues/efficiencies related to funding. |
| Materials & Equipment | Definition: Supplies are available to implement and deliver FIM programs. |
| Access to Knowledge & Information | Definition: Guidance and/or training is accessible to implement and deliver FIM programs. Program is already being implemented, or it has been decided to implement a certain FIM model (i.e., the model was adopted), but the amount of available guidance materials, technical assistance, and/or access to experts about implementing the model is lacking (or abundant), which impacts implementation success.  *Note: This differs from Evidence-Base in that they are not deciding to engage in FAM or choose a model, and needing to find evidence to support that decision, they already have their model, it is just hard to implement due to a lack of guidance.* |
| **Individuals Domain**    Definition: Use codes from Individuals domain whenever interview participants talk about specific people or populations – across any domain – that either implement, benefit from, or otherwise influence the FIM intervention. Use if one of the Roles codes applies **and** one of the Characteristics codes applies. For each person or population discussed, both a Roles and Characteristics code should be applied. | |
| *Roles Subdomain* | |
| Leadership | Definition: Individuals with a high level of authority, including key decision-makers, executive leaders, or directors. Leadership at Elevance, Feeding America, each Food Bank, and each Healthcare partner. These are executive level leaders who we will likely have little interaction with directly. They oversee most of the operations of their respective organizations. They have authority to approve FIM initiatives but have very little day-to-day interactions with FIM teams or activities. |
| FIM Deliverers | Definition: Individuals who are directly or indirectly involved with planning or delivering FIM programs. These are members of the food bank and healthcare partners who help implement the program FIM program. |
| FIM Recipients | Definition: Individuals who are directly or indirectly receiving FAM programs. These are the patients/neighbors who are screened, referred, and/or receive FIM services. |
| **Characteristics Subdomain** | |
| Need | Definition: The individual(s) has deficits related to survival, well-being, or personal fulfillment, which will be addressed by implementation and/or delivery of FIM programs.    Example: Access to affordable diabetic friendly food was lacking in our area, and so there was a real need among our clients for our medically tailored meals program which contributed to high buy-in for the program. |
| Capability | Definition: The individual(s) has interpersonal competence, knowledge, and skills to fulfill role.    Example: We hired someone to manage our FIM program who had extensive expertise due to filling a similar role at another larger food bank. They were able to hit the ground running and ramp up the program quickly. |
| Opportunity | Definition: The individual(s) has availability, scope, and power to fulfill role.    Example: Our manager of the FIM project was stretched thin trying to fulfill several roles by themselves until we could hire more help, which limited the scope of our project. |
| Motivation | Definition: The individual(s) is committed to fulfilling role.    Example: The lead for the FIM work for our healthcare partner is very engaged and passionate about FIM work and always attends meetings and advocates for this work with their leadership. |
| **Implementation Process Domain**    Definition: The act of implementing and evaluating FIM includes a set of processes and procedures that may be working well or not working well. Use codes from the Implementation Process domain when interview participants are talking about something related to implementing a FIM project that is a clear facilitator or barrier. | |
| Teaming | Definition: Joined together, intentionally coordinating and collaborating on interdependent tasks, to implement FIM programs.    Example: Before starting our FIM project, we formed a FIM team to help us plan, organize, and manage the FIM project. |
| Assessing Needs | *Note: Use this construct to capture themes related to Assessing Needs that are not included in subconstructs.* |
| FIM Deliverers | Definition: Collect information about the priorities, preferences, and needs of deliverers to guide the implementation and delivery of FAM programs.    Example: Before developing our FIM model, we held a focus group with staff that would be involved in delivering the program to ensure workflows would be smooth and the project met their needs. |
| FIM Recipients | Definition: Collect information about the priorities, preferences, and needs of recipients to guide the implementation and delivery of FIM programs.    Example: We surveyed people who screened positive for food insecurity at the healthcare site to see what they want in terms of services to address food insecurity. |
| Assessing Context | Definition: Collect information to identify and appraise barriers and facilitators to implementation and delivery of FIM programs.  Example: We conducted a Strengths, Weaknesses, Opportunities, and Threats, analysis prior to developing our FIM model to ensure it aligned with our strengths and weaknesses. |
| Planning | Definition: Identify roles and responsibilities, outline specific steps and milestones, and define goals and measures for implementation success in advance.    Example: We developed a comprehensive action plan that guided the implementation of our FIM program. |
| Implementation Strategies | Definition: Choose and operationalize implementation strategies to address barriers, leverage facilitators, and fit context.  Example: We offered FIM training but realized it didn’t cover how to load boxes to achieve certain nutritional standards, so we revised the training and retrained the staff. |
| Engaging | *Note: Use this construct to capture themes related to Engaging that are not included in subconstructs.* |
| FIM Deliverers | Definition: Attract and encourage deliverers to serve on the implementation team and/or to deliver FIM programs.    Example: We made sure to include some of our frontline staff, who handle the day-to-day duties of the FIM program to serve on our planning and implementation team for the FIM project. |
| FIM Recipients | Definition: Attract and encourage recipients to serve on the implementation team and/or to deliver FIM programs.    Example: We instituted a client advisory board some years back to provide input on the programs we provide. They reviewed our FIM program plans and we made revisions based on their feedback. |
| Doing | Definition: Implement in small steps, tests, or cycles of change to trial and cumulatively optimize delivery of FIM programs.    Example: Our healthcare partner has 11 sites, but we piloted our FIM program at just one of the sites so we could get a better sense of the flow of participants, work out kinks in the tracking processes, and make tweaks to protocols. After that test run, we expanded to the rest of the sites. |
| Reflecting & Evaluating | *Note: Use this construct to capture themes related to Reflecting & Evaluating that are not included in subconstructs.* |
| Implementation | Definition: Collect and discuss quantitative and qualitive information that indicates the degree to which **implementation outcomes** are achieved. Code only if this information was used to inform or influence program implementation.  *Note: Reach data and any other metrics they use to track what they’re doing/active program delivery.*    Example: This is about evaluating how the FIM program was conducted. |
| Impact | Definition: Collect and discuss quantitative and qualitative information that indicates the degree to which FIM **program outcomes** are achieved. Code only if this information was used to inform or influence program implementation.  *Note: Survey data and clinical data.*    Example: This is about evaluating what effect the FIM program had. |
| Adapting | Definition: Modify the FIM program and/or Inner Setting for optimal fit and integration into work processes.  Example: Based on our FIM evaluation results, we added better systems for tracking referrals and processes to medically tailor food boxes for diabetic and heart healthy patients because we saw these were the two types of patients most referred to us based on the evaluation findings. |
