# Supplementary material for: Barriers and facilitators to implementing Food is Medicine programs: Evidence from 21 food bank–healthcare partnerships
Source: Transl Behav Med. 2025 May 31;15(1):ibaf013. doi: 10.1093/tbm/ibaf013 (PMC12169342; doi:10.1093/tbm/ibaf013)
Supplement: ibaf013_suppl_Supplementary_File_2 [file ibaf013_suppl_supplementary_file_2.docx]

**Supplemental File II**

**Semi-Structured Interview Guide.**

*We are having these calls to help us understand all the grantees’ food as medicine (FAM) efforts in more detail and to learn from you all ways we can be most supportive.*

*First, thinking about your pre-FAM3 food as medicine work, I’d like to ask you a few questions to understand how your food as medicine work started and the path that a patient/neighbor follows from initial screening all the way through receiving services.*

1. What originally got your group interested in the idea of food as medicine?

PROBES:

- How did you come up with your concept for your food as medicine program?
- How did your food bank and healthcare partnership come to be?
- What was the planning process like?
- How did you roll it out, initially?
- Did initial implementation of your food as medicine program go according to plan? (Describe, if so or if not, why?)
- What early factors were key to the successful launch of the project?

2. Could you share an overview of your food as medicine project?

a. How does the food security screening and referral process unfold?

PROBES:

- What else do you screen for, if anything?
- How is that information stored and tracked? Who has access to this information?
- How are referrals communicated to those that need to know?
- Do you have a closed loop referral process? If no, why not? If yes, please explain.
- How are patients/neighbors then connected with needed support/services?
- Are there specific patients you are prioritizing (e.g. people with diabetes)?

b. What support/services are provided and/or are patients being referred to?

PROBES:

- Is there a process for tailoring support/services to meet specific needs? If so, describe.
- For how long does/will each patient/neighbor receive support from this program? During that time, how regularly do patients/neighbors interface with your food as medicine program?
- How is each interaction with food bank or health care staff tracked?
- What are your main challenges around connecting patients/neighbors with services?

c. How would you describe the relationship between the food bank and healthcare partners on this project?

PROBES:

- How do you divide leadership? Roles and responsibilities? Communication/coordination?
- Do you face any data sharing challenges? If so, please explain.
- Are there aspects of this partnership that work well? Are there aspects of this partnership that could be improved?

d. What are you most excited about with your food as medicine work, such as some key successes you’ve had?

*Now we’d like to look forward to this new grant period and your FAM3 project.*

3. What do you most look forward to with your FAM3 project, such as some key goals you want to accomplish?

PROBES:

- We talked earlier about the screening and referral process, what if any changes are you planning for your FAM3 project?
- What if any changes to engagement with patients/neighbors are you planning for your FAM3 project?
- What are some other areas in which you’d like to change or improve for your FAM3 project?
- What are some challenges you anticipate in reaching your goals and/or implementing changes?
- Is your FAM3 project building on any lessons learned from previous projects? How?
- How prepared is your team/partners to implement your FAM3 project?
- What information, resources, or technical support do you already know you would like to receive to help implement your FAM3 project?

4. Could you describe any evaluation work you all have been doing with your previous food as medicine work?

PROBES:

- What have you been measuring and how? What frequency? Who is responsible for collecting what types of data?
- What would you like to be measuring for your FAM3 project that you aren’t currently?
- What are the main barriers to your planned data collection for your FAM3 project?
- Has your team considered assessing impacts using clinical data?
- If no: Why not?
- If yes, but not done: What is needed to make that happen?
- If yes and done: What are you currently measuring? Do you plan to continue for FAM3?
- Who has been leading and who has been involved in your evaluation work? Will they be involved in your FAM3 project?
- How would you describe your in-house evaluation/research capacity?
- If high capacity: What has worked well in the past for your team to work with external evaluators? What hasn’t?
- If lower capacity: What are some areas you feel your team will need the most support in?

5. Is there anything else you would like to share with us?
